# Can Commercial Digital Cameras Be Used as Multispectral Sensors? A Crop Monitoring Test

**DOI:** 10.3390/s8117300

**Published:** 2008-11-17

**Authors:** Valentine Lebourgeois, Agnès Bégué, Sylvain Labbé, Benjamin Mallavan, Laurent Prévot, Bruno Roux

**Affiliations:** 1 CIRAD UPR SCA, Station La Bretagne, Saint-Denis, La Réunion, F-97408 France; 2 CIRAD UMR TETIS, Maison de la Télédétection, Montpellier, F-34093 France; E-Mails: agnes.begue@cirad.fr; benjamin.mallavan@teledetection.fr; 3 Cemagref UMR TETIS, Maison de la Télédétection, Montpellier, F-34093 France; E-Mail: sylvain.labbe@cemagref.fr; 4 INRA UMR LISAH, SupAgro, Montpellier, F-34060 France; E-Mail: laurent.prevot@supagro.inra.fr; 5 Avion Jaune, Minéa Incubation, Montpellier, F-34196 France; E-Mail: bruno.roux@lavionjaune.fr

**Keywords:** Digital camera, spectral sensitivity, vignetting, radiometric correction, crop monitoring, airborne images

## Abstract

The use of consumer digital cameras or webcams to characterize and monitor different features has become prevalent in various domains, especially in environmental applications. Despite some promising results, such digital camera systems generally suffer from signal aberrations due to the on-board image processing systems and thus offer limited quantitative data acquisition capability. The objective of this study was to test a series of radiometric corrections having the potential to reduce radiometric distortions linked to camera optics and environmental conditions, and to quantify the effects of these corrections on our ability to monitor crop variables. In 2007, we conducted a five-month experiment on sugarcane trial plots using original RGB and modified RGB (Red-Edge and NIR) cameras fitted onto a light aircraft. The camera settings were kept unchanged throughout the acquisition period and the images were recorded in JPEG and RAW formats. These images were corrected to eliminate the vignetting effect, and normalized between acquisition dates. Our results suggest that 1) the use of unprocessed image data did not improve the results of image analyses; 2) vignetting had a significant effect, especially for the modified camera, and 3) normalized vegetation indices calculated with vignetting-corrected images were sufficient to correct for scene illumination conditions. These results are discussed in the light of the experimental protocol and recommendations are made for the use of these versatile systems for quantitative remote sensing of terrestrial surfaces.

## 1. Introduction

Recent developments in sensor technologies have made consumer digital cameras more and more efficient and affordable. The main advantage of digital photography lies in simplified image processing. The use of digital cameras or webcams has appeared across multiple different domains, including colorimetric applications [1-3] and environmental applications by characterizing and monitoring features [4-5]. Agricultural applications benefit significantly from the use of digital cameras in plant phenology monitoring [6-8], precision farming [8-10], production assessment [9, 11-12], and vegetation structure characterization using hemispherical lenses [13-14]. Digital cameras can be used either in a stationary installation [6-7] or onboard a light aircraft or unmanned aerial vehicle, a deployment which is made possible thanks to their low weight [15-16]. In most cases, the digital photographs are recorded in JPEG or TIFF formats, and the Red, Green and Blue (RGB) channels are obtained through simple image processing. The RGB channels are then either used for image classification, or combined in spectral indices to be correlated with the surface parameters of interest. Despite a number of interesting results, these digital camera systems generally suffer from signal distortions due to the on-board image processing algorithms, and they offer limited quantitative data acquisition capabilities.

**Figure 1.**
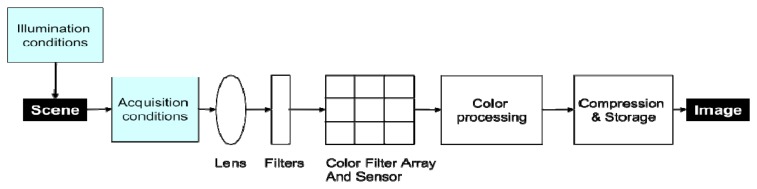
Environment-dependent (blue boxes) and camera-related (white boxes) factors involved in the image acquisition process.

Several factors affect the signal, and the conversion between object luminance and digital image measurement is not straightforward (Figure 1). These factors are camera-related (color processing algorithms, camera settings and vignetting) and environment-dependent (sun geometry, atmosphere and flight altitude). Although researchers have paid significant attention to image geometry [17], to date much less attention has been paid to the relation between pixel values and target radiance [14]. In certain cases, like time series analysis or quantification of surface parameters, pixel radiometry must be corrected in order to be comparable across time and space. The main sources of radiometric distortions are:

Gamma correction: Digital cameras are based on CCD (charge-coupled device) or CMOS (Complementary Metal Oxyde Semiconductor) detectors that are linear photoconductive devices. If twice the flux of photons is received on a given pixel, twice the output value will be generated. Despite the intrinsic linearity of these sensors, digital value output from standard digital images is not a linear measure of object brightness, since the image processing software embedded in digital cameras is designed to emulate the non-linear behavior of the human eye. Accordingly, most modern consumer cameras use some type of gamma adjustment to map the image to the available quantization range in order to improve esthetics [18].

CFA interpolation (or demosaicing): Most of the digital cameras use a single sensor with a color filter array (CFA) that permits only one color to be measured at each pixel (Red, Blue or Green, sometimes Cyan). To create the color image, the missing color values are estimated for each pixel by means of CFA interpolation. The color interpolation process, also known as demosaicing, is generally achieved using a proprietary algorithm.

Vignetting: This distortion refers to the phenomenon of brightness attenuation away from the image center, and is an artifact that is prevalent in digital photography. Several mechanisms may be responsible for vignetting effects. Some arise from the optical properties of camera lenses, the most prominent of which is off-axis illumination falloff or the cos^4^ law. Other sources of vignetting are geometric in nature, including the light arriving at oblique angles to the optical axis and subsequently being partially obstructed by the field stop or lens rim [19]. Although lens manufacturers attempt to design their lenses so as to minimize the effects of vignetting, it is still present to some degrees in all lenses. Vignetting presents problems in measurement applications when radiometric quantities are estimated from images [20].

Radiometric normalization between images: The camera measures radiance, the energy reflected by the scene. This radiance depends on the incident radiation at the time the image was acquired, and on the optical properties of the scene (Figure 1). The quantity and quality (spectral composition) of the incident radiation is related to the solar zenith angle and to atmospheric conditions. The radiance is converted into Digital Numbers (DN) depending on the camera settings (the lens f-stop, the exposure time, and the gain via the ISO setting). To compare images, one must take into account both the incident radiation and the camera settings.

We observed that many factors play a role in image acquisition: built-in gamma correction and image interpolation, vignetting, camera settings, and quality and quantity of incoming radiation conditions. The printing industry and medical communities have investigated the properties of consumer digital cameras as colorimetric measurement device [3, 21, 22]. However, few studies have investigated the utility of this type of camera as a multispectral radiometer, especially for out-door measurements. The objective of this paper was to test simple radiometric corrections of the aforementioned camera-related distortions and environmental conditions, and to quantify the corrections in terms of ability to better monitor vegetation variables. Our algorithm employed three steps: 1) correction of the camera-related factors, 2) correction of the environment-dependant factors and 3) quantification of the signal correction in terms of vegetation variables monitoring.

To achieve this, in 2007 we conducted a five-month experiment in which we flew over sugarcane trial plots using original RGB and modified RGB (Red-Edge and NIR) cameras loaded onto an ultralight aircraft. Our hypothesis was that if accurate radiometric data could be derived from the images using simple post-processing, we could design a cost-effective imaging system that could lead to exciting possibilities for new approaches in precision farming.

## 2. Data acquisition

Our radiometric data acquisition system consisted of an ultralight aircraft equipped with modified digital cameras that acquired and measured the sunlight reflected in five different spectral bands. Between May and September 2007, seven airborne flight trials were conducted over a 7.3 ha experimental sugarcane field on Reunion Island, a French territory that lies in the Indian Ocean.

### 2.1. Digital cameras

**Figure 2.**
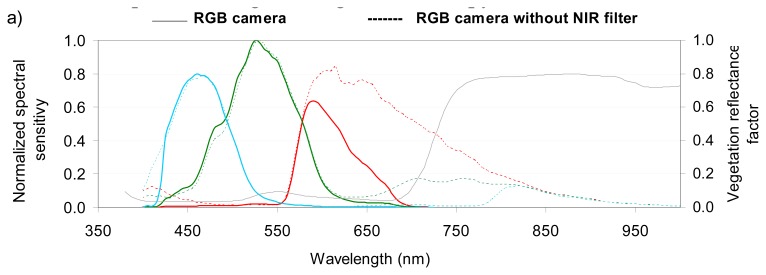

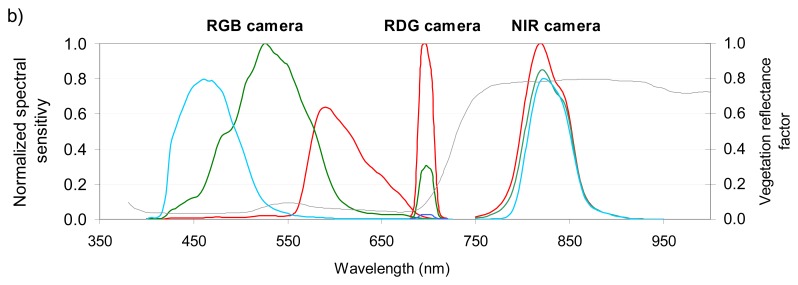
Normalized spectral sensitivity of CANON EOS 400D cameras: **(a)** the original (RGB) and modified (without NIR filter), **(b)** the original (RGB) and modified (RDG and NIR). The colours of the lines correspond to the camera channels. The grey line is a standard reflectance profile of a green vegetation canopy.

We used three Canon® EOS 400D digital cameras (10.1 Megapixel CMOS sensor); each of which had a focal length of 35 mm. The spectral sensitivity of the three cameras was measured in the laboratory with a monochromatic source 1.2 nm wide [23-24]. The original camera measured radiation in Red, Green and Blue spectral bands (Figure 2a), and is hereafter referred to as the RGB camera. The other two cameras were modified to allow them to detect radiation in additional spectral bands (Figure 2b). The modification consisted of removing the original internal NIR high-pass filter (Figure 2a) and adding specific band-pass filters [8, 15]. One camera was then equipped with an external band-pass Oriel filter (690-709 nm 50% cut), and the other with a LDP LLC XNiteBPG filter (808-855 nm 50% cut); these cameras are hereafter referred to as the RDG (Red-edge) and the NIR (Near Infrared) cameras. Figure 2b shows that the wavebands are broad for the RGB camera, and especially narrow for the RDG camera. If we consider the standard spectral profile of a green vegetation canopy, our results also indicate that the RDG is located at the beginning of the slope between the red and near infrared spectral domains.

The camera focus was set to infinite. The settings of the three cameras (aperture, shutterspeed, and sensitivity, Table 1) were determined by flight acquisition tests, and were adjusted manually to eliminate saturated values in any band. The settings were kept unchanged throughout the experiment.

### 2.2. Airborne image acquisition

The three multispectral cameras (2.8 kg) were assembled and mounted on board an ultralight aircraft [24]. The cameras were pointed in the same direction (vertical viewing). Their shutters were synchronized to a single trigger. The ultralight aircraft has a slow flight speed (apparent ground speed between 50 km/h and 70 km/h, depending on wind conditions) that does not result in motion blur when images are taken at a low altitude (600 m). The position of the aircraft was recorded by a GPS data unit during each flight.

Seven flights were undertaken over the La Mare site between May and September 2007: May 2, May 25, June 8, June 29, July 23, August 13, and September 5. The flights were performed between 11:00 am and 12:00 noon solar time, on clear days. The aircraft flew transects over the field at about 600 m altitude, producing images with a ground resolution of between 10 and 12 cm.

During the same period, additional airborne acquisitions were taken from other agricultural regions of the island at different altitudes (between 300 m and 1,500 m), leading to a total of about 500 photographic images per camera acquired under similar atmospheric conditions (clear days) and irradiance geometric parameters (around 12:00 solar hour).

All the images were recorded in JPEG and RAW (termed CR2 for CANON cameras) formats.

### 2.3. The ground experiment

**Figure 3.**
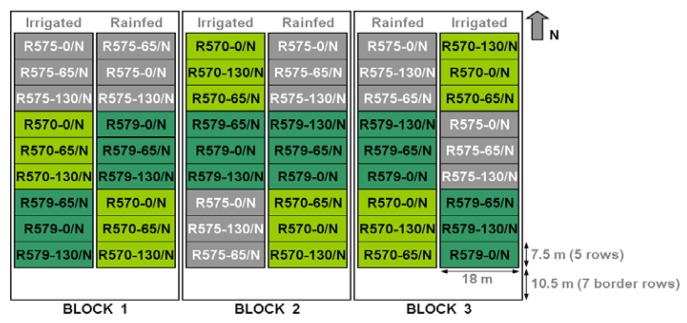
Layout of the La Mare 2007 experimental trials: irrigated/rainfed, three sugarcane cultivars (R570, R575, R579), and three nutrient inputs (0N, 65N, 130N), three replications each.

The sugarcane experimental field is located in La Mare, Sainte Marie (Lat 20.9°S; Lon 55.5°E), in the northern part of Reunion Island (average altitude of 60 m). The field was divided into three randomised blocks cultivated with three cultivars of sugarcane (R570, R575, R579) under three different nitrogen inputs (0/N, 65/N, 130/N) and two water treatments (irrigated and rainfed) (Figure 3). For each combination of treatments (cultivar, nitrogen, irrigation), the size of the plot in each block was 135 m². Each comprised 5 rows 18 m in length, with a 1.5 m inter-row separation.

The sugarcane field was in its seventh month of growth at the beginning of the experiment in May, and exhibited a closed canopy at that time. Plant measurements were performed monthly on the R570 and R575 cultivar plots, including Leaf Area Index (LAI) and leaf greenness (SPAD, SPAD-502 MINOLTA). Values of LAI and SPAD were obtained using non-destructive measurements on the three central rows of each plot in order to avoid border effects, and were averaged for each plot [25]. An integrated index, the canopy chlorophyll content CC, was estimated from the following equation:

(1) CC=SPAD∗LAI

The CC index is linked to NDVI [26, 27]. The mean annual precipitation in the study area was 1514 mm/year, but the experiment took place during the dry season (alternating dry and wet periods).

## 3. Data processing

The data processing steps aimed to recover comparable values of crop variables across both space and time. The sources of radiometric distortions in the measurements, derived both from the camera itself and from the acquisition conditions, were listed in the introduction. The radiometric correction process implemented in this study included the following stages:

- Decoding unprocessed digital photo;
- Correcting the vignetting;
- Normalizing the image series over time.

We then calculated the relationship between image spectral values and ground measurements.

### 3.1. Decoding unprocessed digital photos

**Figure 4.**
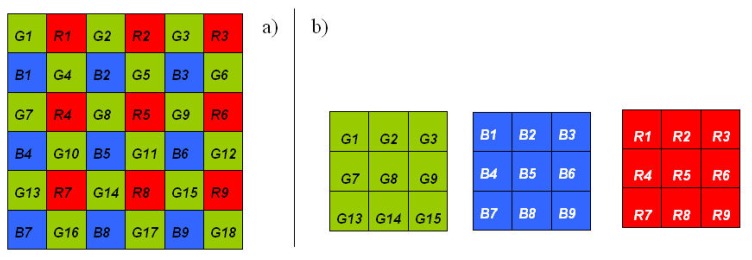
**(a)** Color Frame Array of the Canon EOS 400D and **(b)** extraction of “spectrally pure” images.

The CANON cameras produce their images by means of a Bayer matrix (or Bayer CFA) whereby each individual pixel is filtered and coded as red (R), green (G) or blue (B) (Figure 4). As explained in the introduction, for most commercial cameras, a “black box” proprietary interpolation algorithm is applied to the Bayer matrix to calculate a full frame RGB image which simulates a realistic appearance for the human eye. The image radiometric resolution is often lowered during this operation (generally from 12 to 8 bits) and compressed (using JPEG format). In order to preserve the information contained in the image, we worked with the unprocessed CMOS data files (CR2 format for CANON cameras). To decode these unprocessed images, for which the camera manufacturer does not always provide decoding software, we used IRIS 5.5, a free software package available on the web (Buil C., personal communication; http://www.astrosurf.com/buil/iris/iris.htm). This software is based on a portable open source program, *dcraw* (Coffin D., personal communication; http://www.cybercom.net/∼dcoffin/dcraw/) which supports most RAW formats and is available on most operating systems. IRIS splits the CR2 files into four monospectral images, each corresponding to a spectral band (G, G, R, B). The resulting output images (1,953 × 1,301 pixels) are four times smaller than the originals. [13] who also used the *dcraw* program, demonstrated perfect linearity between the DN of decoded images and quantum sensor measurements.

For the RGB camera, we kept the Red image, one of the two Green images, and the Blue image. Only the Red image extracted from the CFA was stored for the RDG and NIR cameras, as the Red band has the best signal to noise ratio (Figure 2b). This process resulted in five spectral bands (Red, Green, Blue, Red-Edge, NIR) for each shot.

The difference between the unprocessed images and JPEG images was studied by comparing their respective DN values. Because of the intrinsic nature of the JPEG and unprocessed formats, and the difference in image size (JPEG: 3,888 × 2,592 pixels - RAW: 3,906 × 2,602 pixels), it was not possible to compare the images on a pixel-by-pixel basis. Consequently, we made the comparison on a set of training polygons representing a large range of radiometric values: dark road, bright flat roofs, dense vegetation, …etc. The polygons were selected in homogeneous areas to limit location uncertainty. The test was done on the three spectral bands (Red, Green, NIR) of an image acquired on June 29.

### 3.2. Vignetting correction

The correction of the image vignetting was performed using three steps: preparation of the data set, calculation of the radiometric distortion by model fitting, and the correction itself. The calculations were made separately for each spectral band.

To determine the vignetting effects in an image, the most straightforward approach involves capturing an image that spans a uniform scene region, such that brightness variations can be attributed solely to vignetting [28]. However, obtaining suitable imaging conditions for this approach can be challenging, and measurements are valid only for images captured by a single camera using identical settings [19]. We thus chose to calculate for each spectral band an average image from the 500 images acquired during the whole experiment over all different sites and at different altitudes. In order to avoid radiometric artifacts, we thresholded the DN values so as not to include pixels with a very high signal (buildings for example). Specific thresholds were applied to each spectral band. We ultimately subtracted from the mean image the dark current (DN = 255) added by the IRIS software during the decoding step. The resulting five mean images (Red, Green, Blue, Red-Edge, NIR) were then assumed to be directly proportional to luminance and were used to calculate and model the 2D radiometric profiles.

Most methods for vignetting correction use a parametric model to simplify estimation and minimize the influence of image noise [19]. Empirical models such as polynomial functions and hyperbolic cosine functions are typically used. We chose to fit the radiometric profile by using least square mapping to a bidimensional polynomial, resulting in a smoothed approximation of the vignetting effect. On our mean images, we tested different polynomial orders, from 2 to 7, to fit the optical deformation. Ultimately this polynomial function was used to create a filter mask that was applied (in a multiplicative way) to each image in order to eliminate vignetting.

Assuming that illumination conditions could cause vignetting-like effects (for instance “hot spot” effects) this process was initially performed separately for each date. In our experiment, this approach did not improve correction results (results not shown), so we chose to average a larger number of images that covered all dates in our study (about 500 images) to derive the vignetting profile.

### 3.3. Radiometric normalization

Radiometric normalization consists of rendering images acquired under different irradiance conditions and with different cameras that are comparable in terms of DN. By normalization, we mean here that neither absolute calibration coefficient nor incident radiation measurement was available.

The simplest and most common normalization method involves calculating normalized brightness for the RGB channels. This is performed for each image by dividing the brightness value for each of these channels by the total brightness of the image [6-7]. This method is satisfactory because it succeeds for acquisitions made with different irradiance and camera settings. However, despite its effectiveness, residual variations can be attributed to differences in the spectral distribution of incident solar radiation which are linked to the fraction of diffuse radiation in the total incident light [7]. The same advantages and limitations play a role when a spectral vegetation index is calculated [6-9]. Another empirical method, often used with satellite images, consists of using invariant dark and bright points in the image [29-30]. These points can either be invariant scene features like roads, parking areas or buildings [15], or experimental targets like colored panels.

We tested two methods of radiometric normalization: normalization with invariant targets, and normalization using the cosine of the solar zenith angle:

- Six invariant targets were selected by photo-interpretation (three types of soil, a road, a bush and a building). Polygons were used to extract the DN in the five bands across all the images from the time series. Subsequently, we chose the June 29^th^ image as our reference for the normalization process. For each spectral band, we calculated a transfer function between the DN of the invariant targets on the reference image and the other dates.
- In the absence of a global radiation measurement, we approximated the global radiation from the cosine of the sun's zenith angle (between 28.6° and 45.5° during the experiment). This method was possible only because the settings of the cameras did not change during our study.

The invariant and cosine normalization methods were validated using four plastic panels ranging in color from white to dark grey (1 × 1 m², about 4 × 4 pixels at 600 m flight altitude after image decoding). They were installed on the ground at each spectral acquisition date, close to the La Mare experimental field.

### 3.4. Relationship with surface parameters

To link the spectral and vegetation measurements, we first had to conduct some image postprocessing: geometric correction, extraction of values related to the experimental plots, calculation of spectral indices, and ground data interpolation.

Referenced to a metric camera, the CANON camera lens distortion was measured as less than one pixel (Pierrot-Deseilligny M., 2008; personal communication), and so no correction was applied. For each set of images (RGB, RDG and NIR) acquired simultaneously using the trigger, we first co-registered the different bands to each other. We then performed a geometric correction on each date using a reference image chosen from the data set (June 29^th^).

The radiometric average value for each band was calculated at the plot scale. This calculation was made using the boundary map of the experimental field, after applying a negative buffer of two pixels in order to eliminate mixed border pixels and avoid possible errors due to the geometric correction.

From these mean values, and for each plot, we derived three normalized vegetation indices.

The Normalised Difference Vegetation Index (NDVI [31]):

(1) NDVI=(NIR−R)/(NIR+R)

The Green Normalized Difference Vegetation Index (GNDVI [32]):

(2) GNDVI=(NIR−G)/(NIR+G)

The Normalized Difference of the Green and Red bands (VIgreen [33]):

(3) VIgreen=(G−R)/(G+R)

where NIR, R and G stand for DN in the Near-infrared, Red and Green bands respectively.

As plant measurements were not taken on the same dates as the airborne acquisitions, a linear interpolation between two ground measurement dates was applied in order to estimate LAI and SPAD values on the required image acquisition dates [25]. We then related the three vegetation indices to the CC (Chlorophyll Content) values and fitted regression functions using the entire data set.

## 4. Results

### 4.1. Airborne images

**Figure 5.**
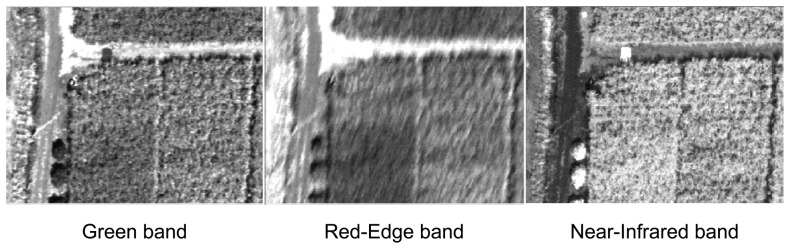
Subset of spectral images acquired with the three cameras (June 29).

The images acquired from the ultra-light aircraft (ULA) were generally neat, with good contrast in the visible and NIR bands (Figure 5). The quality of the Red-Edge images was less satisfactory with a fuzzy rendering (motion blur) certainly due to the combined effects of exposure time and aircraft vibrations.

**Figure 6.**
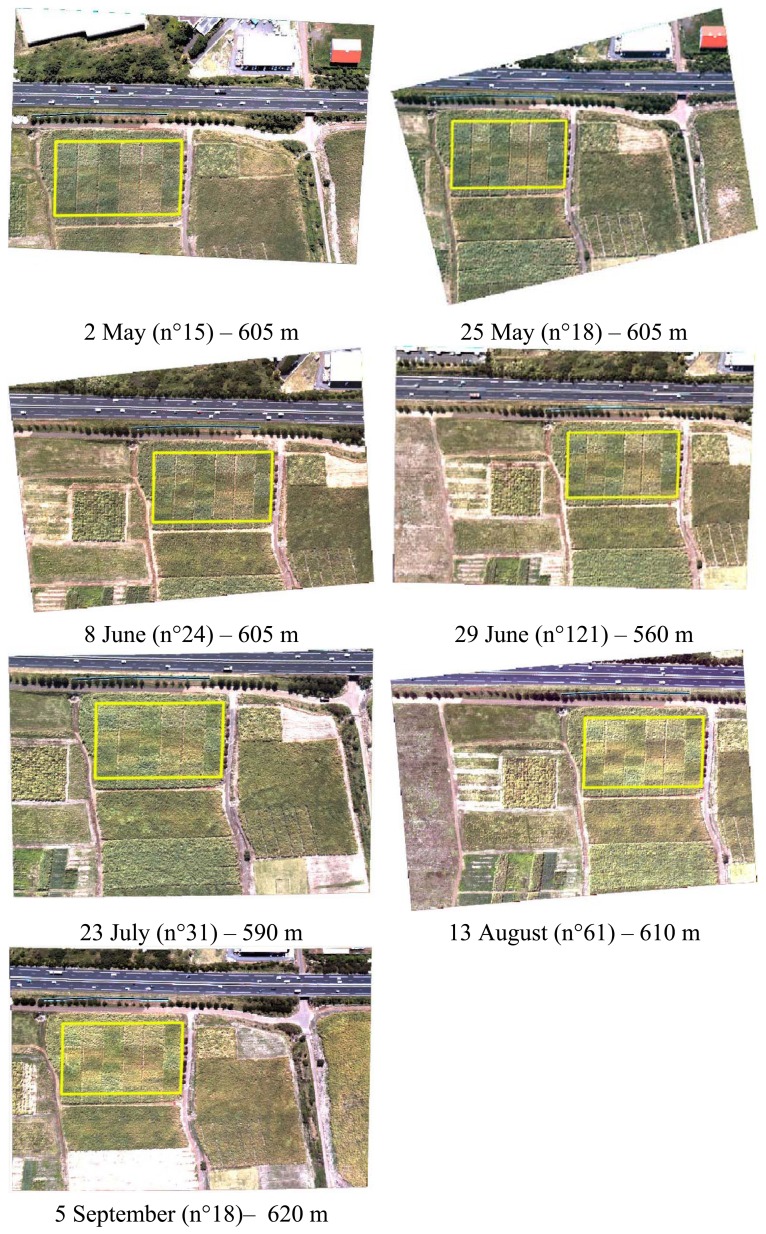
“La Mare” time series of visible images (BGR) taken at an aircraft altitude between 560 m and 620 m. The experimental field is identified by a yellow boundary.

The RGB image time series over the La Mare experimental site is shown in Figure 6. One can see that the sugarcane canopy is fully developed, and that the main change between the dates is in canopy color. The global yellowing of the canopy during the experiment is due to the senescence of the vegetation. Color variability within the experimental field (mosaic of experimental plots) is caused by the different cultivars, irrigation and nutrient treatments.

### 4.2. Radiometric corrections

#### Decoding the unprocessed digital photos

**Figure 7.**
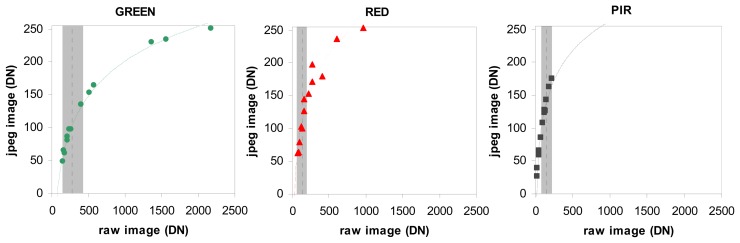
Comparison of the digital numbers (DN) of an image in unprocessed (RAW) and JPEG formats. Logarithmic curves are drawn for each band. Dotted lines represent the mean raw DN measured on vegetation. Grey zones represent mean ± 2 standard-deviations.

We compared the unprocessed (RAW) and JPEG images on a polygon basis. The relationships between the digital numbers from the unprocessed and JPEG versions of one image are shown in Figure 7 for both visible and NIR bands. First, one observes a difference in value depths (8-bits image for JPEG, 12-bits for unprocessed), except for the NIR unprocessed image which suffered from underexposure. Secondly, the relation is not linear and the tonal mapping can be modelled with a logarithmic function.

As shown previously in Figure 2, the spectral response of vegetation is low in the visible bands and high in the NIR band. However, in our case the NIR images were underexposed and produced low DN values. This is confirmed by the low values of the mean DN measured on raw images obtained on La Mare sugarcane trial in the Green (276 ±66), Red (131 ±35), and NIR (153 ±33) bands during the experiment. Thus, one can consider that the relation between Green, Red and PIR bands of RAW and JPEG images was linear for the range of radiometric values observed during crop monitoring (Figure 7).

#### Vignetting correction

**Figure 8.**
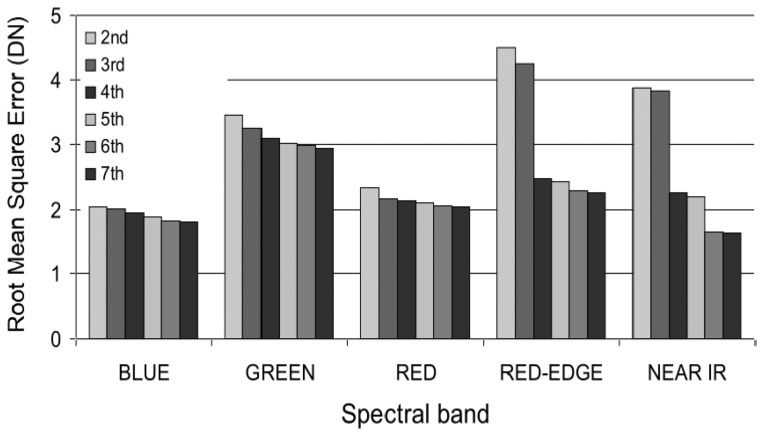
Effect of the polynomial degree of the vignetting function on the quality of the fitted model in each spectral band.

**Figure 9.**
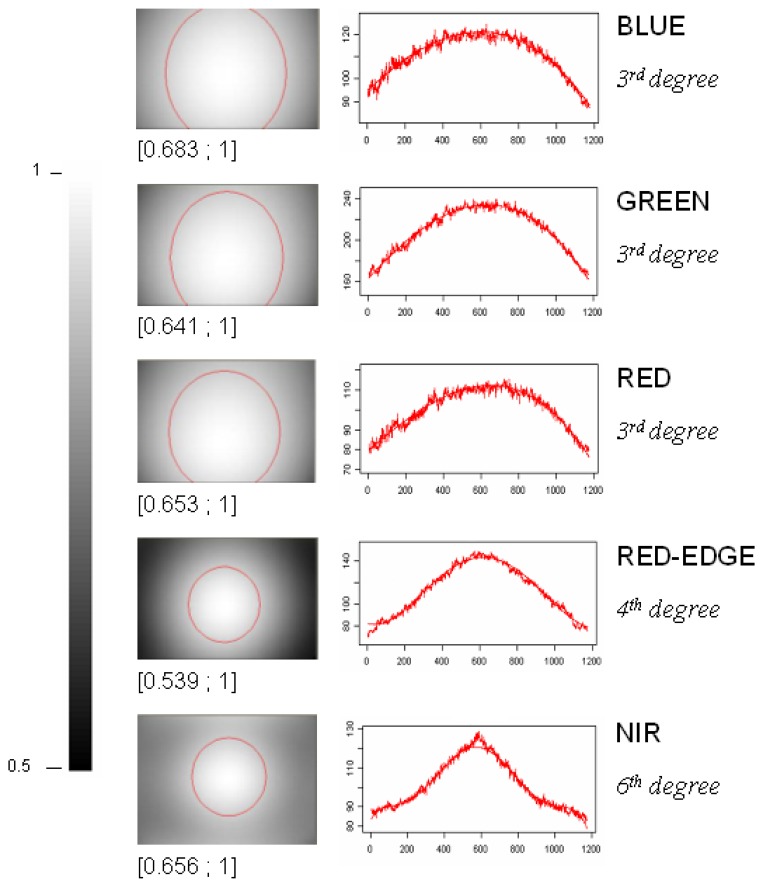
Vignetting filters for the Visible, RDG and NIR images (the circles are iso-contours for a signal loss of 10%), and the corresponding fitted diagonal profiles.

For each spectral band, we calculated a vignetting correction filter by fitting a polynomial function distribution onto an average image computed over the whole data set (about 500 images acquired in different locations and at different altitudes). This vignetting distribution function expresses the vignetting factor for a given position in the image as a polynomial function of position (i.e., row and column coordinates). Different degrees of polynomial were tested for each band and the resulting Root Mean Square Errors (RMSE) are as shown in Figure 8. In the visible (R, G, B) bands, an increase in the polynomial degree led to a small decrease in the RMSE. In the RDG and NIR, a significant decrease of the RMSE occurred between the 3^rd^ and 4^th^ degree. A second decrease between the 5^th^ and 6^th^ degree was observed for the NIR band; the 6^th^ degree polynomial resulted in a good fit at the centre and corners of the image (Figure 9). These results led us to choose a 3^rd^ polynomial degree function to correct the vignetting effects for the RGB, and a 4^th^ and 6^th^ polynomial degree function for the RDG and NIR, respectively.

Once the optimal polynomial function had been found, we produced a vignetting filter image for each spectral band (Figure 9). Analysis of the filters indicated that the images taken with the RGB camera showed similar vignetting profiles, characterized by a smooth decrease in the signal (cosine-type function) as the distance from the centre increased. The signal attenuation was as high as 35% at the corners of the image. In the Red-Edge band the vignetting profile was somewhat sharper, and the loss reached 46% in the corners. The vignetting profile of the NIR images displayed a sharp shape, and attenuation of up to 35%. The shape of the vignetting profile seems more pronounced as the wavelength increases (from the visible to the NIR). Furthermore, in all bands the mask pattern was not symmetrical; recorded maxima were slightly shifted from the centre of the image.

The results obtained with the RGB camera indicate that today's commercial digital cameras are far from perfect, but are still of relatively good optical quality provided the borders of the photo are ignored. This is because vignetting effects are reduced during the manufacturing process. However, when the cameras are modified and equipped with an external pass-band filter, as in the case of the RDG and NIR cameras, the vignetting effects are substantial (large amplitude losses in the RDG, and a sharp profile shape in the NIR).

#### Effect of the vignetting correction on the radiometric stability of targets on one acquisition date

**Figure 10.**
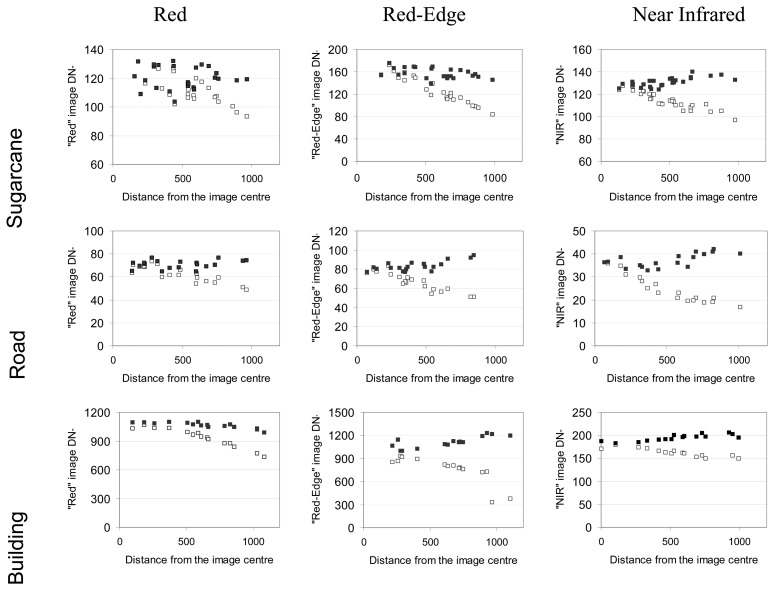
Effect of the vignetting correction on the DN values of three targets (open symbols = before correction; closed symbols = after correction), expressed in distance of the pixel to the centre of the image. The images were acquired on June 29, at different altitudes.

To isolate vignetting effects, we tested the correction on images taken under identical irradiance conditions (same date, around midday), but with changing object positions within the image (different distances from the centre resulting from different framing at different flight altitudes). The effect of the vignetting corrections on the DN of three targets (sugarcane, road, and building) with different spectral properties is shown in Figure 10 for the Red, Near Infrared and Red-Edge bands.

The vignetting effect was visible in all spectral bands and for the three targets, with progressively decreasing DN away from the image centre. The vignetting correction was generally appropriate, yielding a slight over-correction in the NIR. This over-correction does not seem to be linked to the nature of the target. The relative dispersion of the points in all spectral bands can be explained by different flight altitudes, which partly resulted in variable atmospheric noise in the signal. For the sugarcane target, the variability of points in the visible bands (Green and Blue not shown) can be explained by the effects of wind on the canopy (as observed on the images acquired at low altitude). Given these external effects, we consider the vignetting correction to be successful.

#### Effect of the radiometric normalization on the stability of invariant target DNs over time

**Figure 11.**
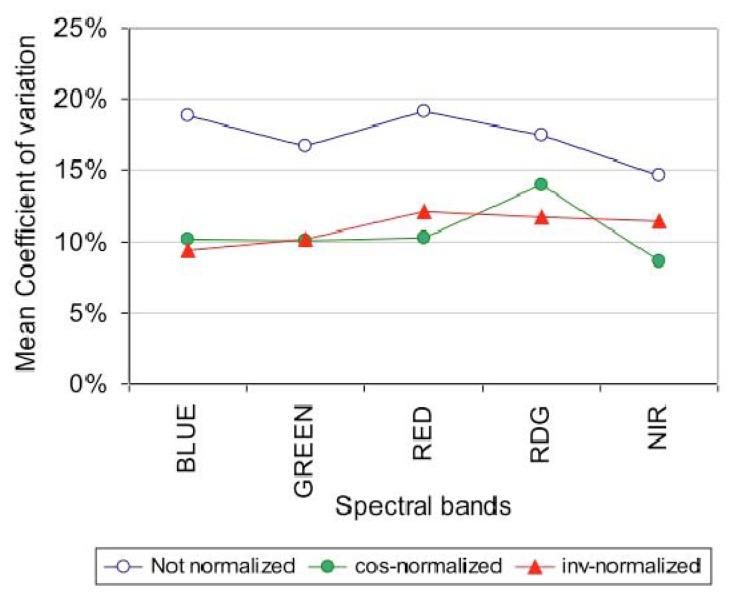
Comparison of different methods of radiometric normalization based on artificial invariant targets: no normalization, cosine and invariant methods. The mean coefficient of variation (CV) is the average of CV of the four artificial targets' digital numbers measured over the acquisition period.

We applied the cosine and invariant normalization methods on a time series of images acquired at 600 m altitude. The effects of these radiometric normalizations on the artificial targets' DNs are shown in Figure 11 for the five spectral bands. The invariant target correction reduced the variations significantly, from a range of variation of [15%-19%] for non-corrected DN, down to a range of variation of [9%-12%]. The cosine correction is globally equivalent to the invariant correction with a variation range of [9%-14%], and it exhibits a slight advantage in the Red and Near-Infrared bands.

**Figure 12.**
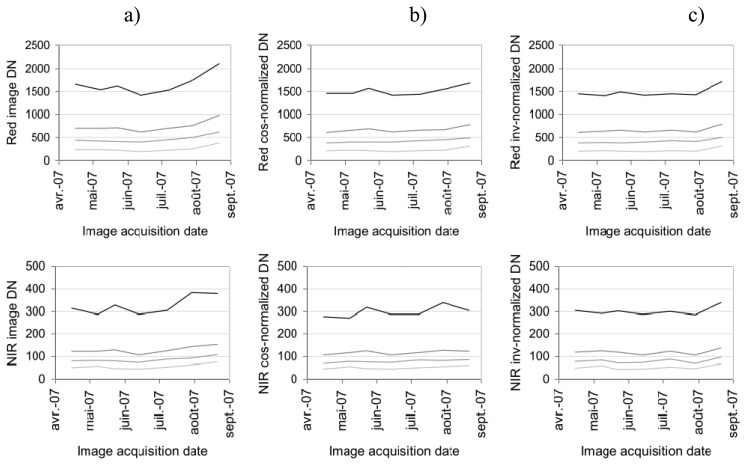
Radiometric values (DN) of artificial invariant target signals in the Red and Near Infrared bands. Comparison of (a) uncorrected values, (b) cosine-corrected values and (c) invariant-corrected values.

When examining the invariant targets in the Red and NIR bands (Figure 12), we observed that bright objects are better corrected in the NIR using the invariant method, but this does not seem to be the case for darker targets. The red-edge radiometric correction is not satisfactory (not shown), presumably because of a location default of the target within the image due to poor image quality.

Overall, the cosine and invariant methods gave similar results. The advantage of the invariant method is to take into account atmospheric variations. The atmosphere is not a first order driving factor in this case as the atmospheric conditions on the acquisition dates were similar (clear days); the first order driving factor is the solar zenith angle.

### 4.3. Effect of the radiometric correction level on the assessment of crop parameters

**Figure 13.**
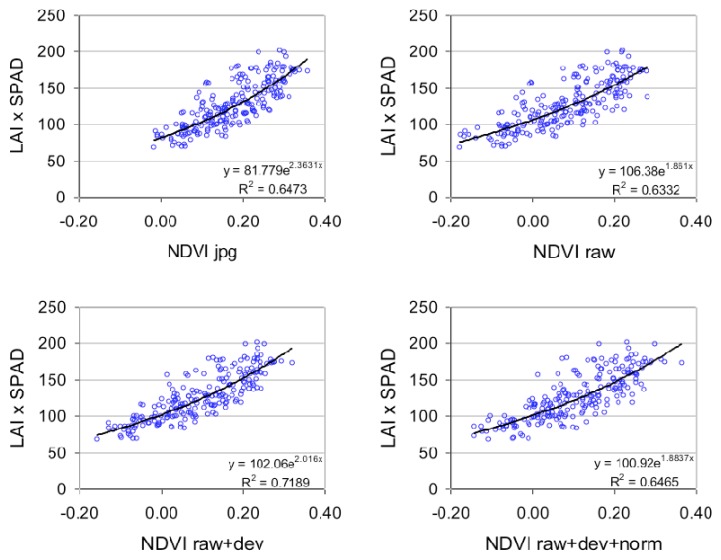
Fitted exponential functions between the Chlorophyll Content (LAI*SPAD) and NDVI for the 36 experimental plots across six dates. NDVI is calculated from the Red and NIR images at different levels of radiometric corrections (jpg = JPEG format; raw = unprocessed format; dev = vignetting-corrected; norm = normalized by invariant targets).

To assess the impact of the radiometric correction on vegetation monitoring, we tested the quality of regressions obtained between NDVI and chlorophyll content (CC). Figure 13 shows four regressions obtained with NDVI calculated for images that are corrected using four different levels of radiometric correction. The images were extracted from the temporal data set acquired over La Mare site in 2007 at 600 m altitude (Figure 6). The four levels of radiometric correction are:

- NDVI_jpg, calculated from the Red and NIR bands extracted from the JPEG images. The JPEG images are downloaded directly from the camera, and the spectral bands are split using image processing software.
- NDVI_raw, calculated from the spectral bands extracted from the decoded unprocessed images.
- NDVI_raw+dev, calculated from spectral bands extracted from the decoded unprocessed images, and corrected from the vignetting effect.
- NDVI_ raw+dev+norm, calculated from spectral bands extracted from the decoded unprocessed images, corrected from the vignetting effect, and normalized over time using the invariant method.

**Figure 14.**
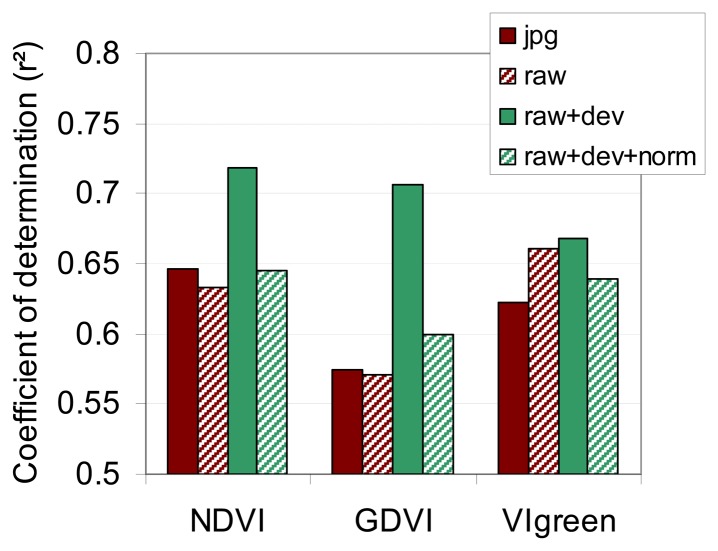
Coefficient of the regression functions between CC (LAI*SPAD) and three vegetation indices (36 experimental plots across six dates). VIs are calculated from the Red, Green and NIR images at different levels of radiometric correction (jpg = JPEG format; raw = unprocessed format; dev = vignetting-corrected; norm = normalized by invariant targets).

As observed in other studies [26], the relationship between NDVI and CC was curvilinear due to NDVI saturation at high LAI values [34]. The point scattering is due to local soil condition variability, and plant measurement inaccuracy (essentially due to the destructive sampling method and the interpolation between dates). Despite the variability, the four regressions can be compared. The key conclusion is that there is no clear advantage between the use of NDVI_jpg, NDVI_raw and NDVI_raw+dev+norm (r² = 0.65, r² = 0.63, and r² = 0.65 respectively). Only the NDVI_raw+dev shows a better relationship with CC (r² = 0.72). We reached the same conclusions when NDVI was replaced by GNDVI (Figure 14), with a more significant effect of the vignetting correction (r² = 0.71 with NDVI_raw+dev, r² between 0.57 and 0.6 for other corrections). When NDVI is replaced by VIgreen (Figure 14), the correction levels were similar, with a slight superiority for the vignetting correction (r² = 0.67).

The absence of an effect from decoding unprocessed images can be explained by two factors. First, the DN values of the vegetation were low in the visible image (strong radiation was absorbed by the vegetation) and in the NIR (resulting from under-exposure of the camera). With DNs of below 200 (Figure 7), the vegetation pixel values were situated in the linear part of the gamma-type correction function embedded in the camera. Second, the DN values were calculated on a polygon basis and therefore the spatial interpolation of the JPEG images did not impact the mean radiometric value. For other types of target, with high radiometric values, our conclusions would be different, and a significant effect of RAW conversion correction would be expected.

In conclusion, the vignetting correction is the only correction that significantly improves the quality of the vegetation indices when a visible (red or green) and a Near Infrared band are used. When two visible bands are incorporated into a vegetation index (VIgreen for example), the vignetting correction is less pronounced. This is because the NIR and visible bands were derived from different cameras with different vignetting functions (Figure 9), while the Red and Green images were acquired using the same optics.

We were surprised to note that the normalization of the spectral bands prior to the calculation of the vegetation index reduced the quality of the relationship. Normalization is a difficult operation with various sources of errors (image registration error, photo-interpretation error, etc.). When individual bands (Red or NIR) are related to vegetation parameters, the relationships are improved by the normalization step (data not shown). In the case of vegetation indices, the radiometric errors of the individual bands are summed, resulting in a very variable result.

## 5. Discussion

In this paper, we tested a simple method of radiometric correction of a series of images acquired over time with three digital consumer cameras onboard a light aircraft. Two out of the three cameras were modified in order to measure radiation in bands other than the default RGB. The quality of the radiometric correction was evaluated against a ground data set of biophysical variables that were independently acquired from sugarcane trials on Reunion Island. The sources of radiometric distortions were both camera-related (image format and vignetting) and environment-dependent (incident radiation).

The modification of digital cameras to allow acquisition in the near infrared band is not new. In 2002, [35] infra-red images were captured using a filter over the camera lens to block energy in the visible bands, and by using the residual sensitivity of the silicon CCD array in near-infrared wavelengths. Today, modern digital cameras are equipped with an efficient infra-red blocking filter that has to be removed before blocking the visible band [8-15]. We showed in this paper that the use of an external band-pass filter allows us to acquire images in any spectral band from 400 nm to 900 nm. However, in that configuration, particular attention must be paid to the camera settings since the energy captured by the CMOS sensor is lower than in the unmodified camera (narrower band or reduced spectral sensitivity in the near-infrared). This leads to a requirement for longer exposure times with the result that data acquisition becomes susceptible to interference as a result of aircraft speed and vibration frequencies.

There are two reasons to use unprocessed images instead of JPEG or TIFF images: JPEG compression is lost and the DNs are not linear with the brightness of the scene. In our case, results show that the image format (JPEG versus unprocessed) has no effect on the correlation between a spectral band (or a vegetation index) and actual surface parameters. This is linked to our range of values for vegetation surface that lies in the linear region of the tone mapping algorithm; this is particularly true for the Near Infrared images that were under-exposed (Figure 7). We did not observe saturation and the JPEG signal was nearly proportional to the unprocessed signal. Furthermore, as we worked at the plot scale with averaged DN values of several pixels, the spatial interpolation due to the JPEG format, and the CFA interpolation, had no visible effect on the signal. However, these conclusions cannot be generalized and we strongly recommend using RAW images instead of JPEG or TIFF.

In respect of image vignetting, the effect was low on the vegetation indices that were calculated with visible bands only, but was high on the vegetation indices calculated with visible and infrared bands like the NDVI. To characterize and subsequently correct the vignetting effects, we developed an original method that is based only on the acquisition data set. Our method has the advantage that it remains usable even when the camera type is unknown or unavailable. However, our method does require a large series of different images with different acquisition and illumination scene conditions. Our results showed that: 1) vignetting is still present in modern RGB digital cameras, and it can be modelled with a second-degree polynomial function, 2) modification of the camera increases the vignetting observed on the images as quantified by [15], and 3) at minimum, a fourth-degree polynomial function is necessary for modelling the vignetting on modified cameras. This spectrally-dependent distortion was responsible for the high sensitivity of the visible-infrared vegetation indices to vignetting.

Ultimately, the radiometric normalization between images still remains a problematic operation. Radiometric normalization using scene invariant targets and linear regression calculations for each spectral band has the advantage that it takes into account variations in incident radiation in each band (this is not the case when using spectral indices [7]. But the invariant method is also more time consuming because it involves additional image processing. Artificial targets generally suffer from insufficient size (often the size is no larger than five pixels) and logistical constraints. Natural targets are generally not invariant, except in the case of bare soil or buildings that are not representative of the vegetation spectral range that we are interested in. In our case, the radiometric normalization using invariant points increased the noise of the vegetation indices because the errors in the Red and Near Infrared bands were cumulative. When working with individual spectral bands, we recommend using the solar zenith angle correction. This simplified radiometric correction is valuable because acquisitions are generally conducted under clear sky and at around midday, and they therefore are captured under similar atmospheric conditions. Furthermore, this method can be used only if the camera settings are manually adjustable and remain unchanged during the experiment. In case of change in camera settings, [36] developed a calibration method for using digital cameras as luminance meters that is independent of exposure settings.

In any case, it is important to characterize the spectral and optical properties of the specific camera used [18]. As only one camera of a specific type has been tested, the conclusions drawn from our experiment are not necessarily valid for other CANON EOS400D cameras or for other camera types.

The next steps in our radiometric correction of time-based image series will be 1) to take into account the spectral variations in radiation due to atmospheric conditions, and 2) to correct for any directional effects [15].

## 6. Conclusions

The use of consumer digital cameras or webcams is increasingly prevalent in environmental applications. The acquisitions are generally performed with automatic settings and the images are saved in JPEG or TIFF formats. Under these conditions, image analysis can be qualitatively satisfying, but the accuracy of the image radiometry is generally too low to permit quantitative estimation of surface parameters.

We showed in this paper that, with a simple procedure, it is possible to increase the radiometric measurement capacity of images acquired by an ultralight aircraft. Putting together several known solutions for radiometric corrections (use of unprocessed images, vignetting correction and radiometric normalization), we showed that a comprehensive image processing workflow was possible for realtime crop monitoring using commercial digital cameras.

The use of modified cameras permits image acquisition in spectral bands that are not currently used in traditional photography, such as NIR, but that are important for accurate surface characterization. Using free software, we read the images in unprocessed camera output format to obtain spectral images that exhibited values close to the true radiance. These spectral images were corrected from the camera vignetting effect using an original method, and were normalized across acquisition dates. The results showed that the Normalized Vegetation Indices calculated from vignetting-corrected images are acceptable indicators for crop monitoring purposes.

In conclusion, for quantitative remote sensing of terrestrial surfaces, the use of commercial digital cameras will increase in the future, thanks to the versatility and multispectral capacities of the available acquisition systems. Their versatility is increased thanks to the flexibility and cost of various lightweight acquisition systems (Ultra-Light Aircraft or Unmanned Aerial Vehicles) that can transport this type of camera. Modifications to camera filters permit narrow-band acquisitions in the visible and in the near-infrared domains; these measurements could be used for example to calculate hyperspectral indices like the PRI (Photochemical Index). However, in cases where bands are too narrow, more investigation is yet required to avoid motion blur due to the speed and vibrations of the aircraft.

## Figures and Tables

**Table 1.** CANON EOS 400D camera settings.

**Camera**	**Shutter speed**	**Sensitivity**	Aperture*

RGB	1/640	100	f-5
RDG	1/160	200	f-5
NIR	1/1000	200	f-5

* f-numbers = diameter of the entrance pupil / effective focal length of the lens
